# Leveraging Computer Vision Face Representation to Understand Human Face Representation

**Published:** 2020

**Authors:** Chaitanya K. Ryali, Xiaotian Wang, Angela J. Yu

**Affiliations:** Department of Computer Science and Engineering, University of California, San Diego La Jolla, CA 92093 USA; Department of Electrical and Computer Engineering, University of California, San Diego La Jolla, CA 92093 USA; Department of Cognitive Science, University of California, San Diego La Jolla, CA 92093 USA

**Keywords:** Face Space, Similarity Judgement, Social Perception, First Impressions, Computer Vision

## Abstract

Face processing plays a critical role in human social life, from differentiating friends from enemies to choosing a life mate. In this work, we leverage various computer vision techniques, combined with human assessments of similarity between pairs of faces, to investigate human face representation. We find that combining a shape- and texture-feature based model (Active Appearance Model) with a particular form of metric learning, not only achieves the best performance in predicting human similarity judgments on held-out data (both compared to other algorithms and to humans), but also performs better or comparable to alternative approaches in modeling human social trait judgment (e.g. trustworthiness, attractiveness) and affective assessment (e.g. happy, angry, sad). This analysis yields several scientific findings: (1) facial similarity judgments rely on a relative small number of facial features (8–12), (2) race- and gender-informative features play a prominent role in similarity perception, (3) similarity-relevant features alone are insufficient to capture human face representation, in particular some affective features missing from similarity judgments are also necessary for constructing the complete psychological face representation.

## Introduction

Face processing is essential to human social cognition, whether recognizing individuals, identifying emotional states, or assessing social traits such as attractiveness and trustworthiness. Having a computational account of how humans psychologically represent faces is essential for developing and testing scientific hypotheses about human face processing, and for developing machine learning and artificial intelligence systems that either socially interact with humans (e.g. social robots) or mediate social interactions among humans (e.g. dating apps and professional network websites)

An implicit assumption in the psychological study of human face processing is the existence of a “face space” (Valentine, 1991), a multidimensional vector space consisting of faces whose vector coordinates correspond to perceived facial properties or features, and the distance between faces determines their perceived similarity. Tools like Multidimensional Scaling (MDS) (Shepard, 1962) have been commonly used to leverage similarity judgments to map (embed) faces into a common vector space representation; such representations have been used to infer mental representations so as to examine perceptual categorization of race (MacLin, Peterson, Hashman, & Flach, 2009), to examine the differences in representation between adults and children (Nishimura, Maurer, & Gao, 2009), and to show that faces rated more typical are located closer to the origin while distinctive faces are farther from the origin (Johnston, Milne, Williams, & Hosie, 1997). Despite its broad use (Dailey, Cottrell, & Busey, 1999; Nestor, Plaut, & Behrmann, 2016; Nishimura et al., 2009; Shepard, 1962; Torgerson, 1965), MDS suffers from several limitations. Notably, the mapping of faces into this embedding space is *abstract*, making it difficult to interpret the features; it is *non-invertible*, offering no easy way to visualize the face corresponding to an arbitrary point in the space; it is *non-generalizable*, such that novel faces not used in the learning of the embedding itself cannot be later projected into the space; it is impractical for assessing the *true dimensionality* of the psychological face space, since training MDS-type algorithms are extremely data-intense.

Separately, computer vision and machine learning techniques have been used to learn to predict (or even manipulate) human judgment of different face attributes, e.g. memorability (Xiao, Oliva, Torralba, & Isola, 2011; Khosla, Bainbridge, Torralba, & Oliva, 2013), trustworthiness, attractiveness, and other social impressions (Song, Li, Atalla, & Cottrell, 2017; Guan, Ryali, & Yu, 2018). However, these work typically do not relate the algorithmic representation of faces to the human face representation, in particular making no attempt to relate distance in the latent representation to human-reported dissimilarity between faces.

Here, we adopt a novel approach, by initializing the face vector space using the latent coordinates of faces generated by different computer vision algorithms, then linearly transforming that vector space such that Euclidean distance in that transformed space recovers human-reported pairwise dissimilarity rating as well as possible – we also include a regularization term that explicitly encourages *efficient* representation. The computer vision algorithms we consider include the Active Appearance Model (AAM) (Cootes, Edwards, & Taylor, 2001), VGG16 (Simonyan & Zisserman, 2014), and an abstract representation obtained through MDS. As we will show, the AAM-based representation not only predicts human similarity judgements on held-out data better than the other models as well as other humans who have assessed similarity of the same face pairs, but also performs best in predicting human social trait (e.g. trustworthiness, attractiveness) and affective judgments (e.g. happy, sad, angry).

Using the AAM-based representation, we then investigate several scientific questions, such as how many facial features are actually involved in human perception of how faces differ from one another, whether features that differentiate demographic groups, in particular race and gender, play an especially prominent role in dissimilarity judgments, and whether similarity judgments utilize features that span the entire psychological face space (or whether there are residual features that cannot be excavated using only similarity judgments).

## Results

**Figure 1:**
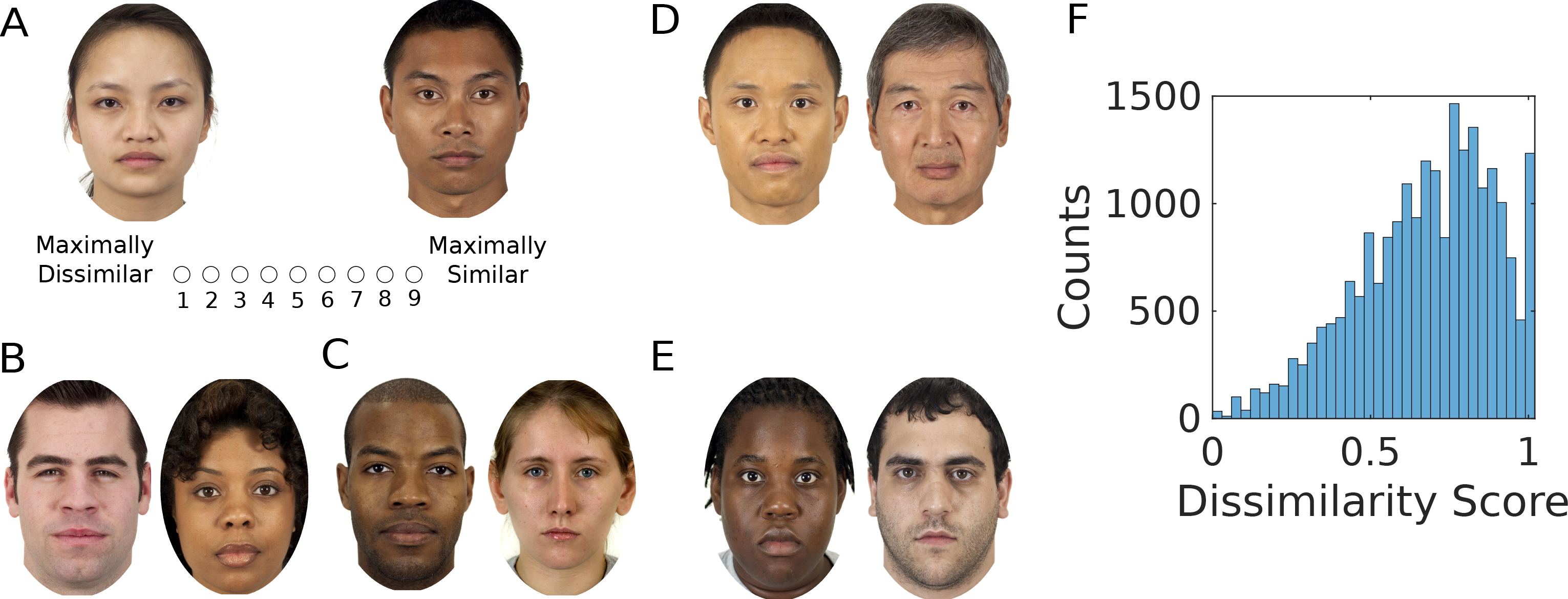
A. Schematic of a trial from data collection. B, C: Low-similarity examples. D, E: High-similarity examples. F. Histogram of empirical dissimilarity scores.

We collected human similarity judgments on pairs of face images through Amazon Mechanical Turk (restricted to participants based in the US). The data set (Ma, Correll, & Wittenbrink, 2015) consists of 595 neutral-expression face images that are gender- and race-balanced (see Methods). Figure 1 shows example image pairs with high and low similarity scores. We find that low-similarity image pairs often differ in race or gender categories, as seen in both low-similarity examples (B, C), while high-similarity pairs can agree on race and gender (D), or not (E). This suggests that human similarity judgments both depend on facial features distinguishing demographic categories and other more subtle structural features.

To model human face representation, we use computer vision models to specify the initial vector space. We first consider AAM (Cootes et al., 2001; Guan et al., 2018; Tzimiropoulos & Pantic, 2013), which computes “shape features”, (*x,y*) coordinates of landmarks that denote invariant parts of faces such as contours of the eyes, eyebrows, nose, mouths, and “texture features”, which are (grayscale) pixel values of each face image warped to have the shape (landmark locations) align with those of the average face in the training dataset. We perform joint principal component analysis (PCA) on the shape and texture features, and retain the first 70 components – as shorthand, we refer to this original AAM space as X. We then linearly transform X so that Euclidean distances between face images are as close to human dissimilarity scores as possible – formally, this is known as metric learning (see Methods).

A simple way of doing metric learning is to linearly re-scale the importance of each feature (basis vector) in X, i.e. humans may weigh different features differently than the computer vision algorithm. However, it may be that humans actually utilize a different set of features altogether. Formally, we enrich our model by allowing the possibility that psychologically relevant features (basis vectors) are linear transformations of the machine vision features (basis vectors), equivalent to first *rotating* the original feature axes, followed by *rescaling* according to psychological importance in similarity judgment – we denote this linear transformation **W**.

Additionally, we consider the possibility that humans are *efficient* in the number of features used to represent faces, which we implement through a *regularization* term in the objective function, by explicitly suppressing the number of basis vectors that significantly contribute to perceptual dissimilarity. Specifically, we penalize the trace of **W**, or the sum of the squared values of the scaling factors (see Methods). In addition, we also consider two more common forms of regularization, based on penalizing the element-wise *ℓ*_1_ and *ℓ*_2_ norms of the transformation matrix (see Methods), which have the undesirable effect of penalizing not only the scaling factors but the amount of rotation allowed before scaling, and not being especially effective at penalizing the scaling factors.

To compare how well different models can capture/predict human similarity perception, we compute the correlation coefficient (c.c.) between model predicted ratings and human dissimilarity scores on held-out face pairs. As a baseline comparison, the average c.c. between one rater’s rating of an image pair and the average rating of the remaining participants on the same image is 0.416. The original AAM representation captures human similarity judgment reasonably well (*r*_test_ = 0.43), and is significantly improved by the linear transformation without regularization (*r*_test_ = 0.532). Further prediction improvement is obtained via all three forms of regularization (*r*_test_ = 0.543 in all cases) on **W**, all of which prevent overfitting to training data.

**Figure 2:**
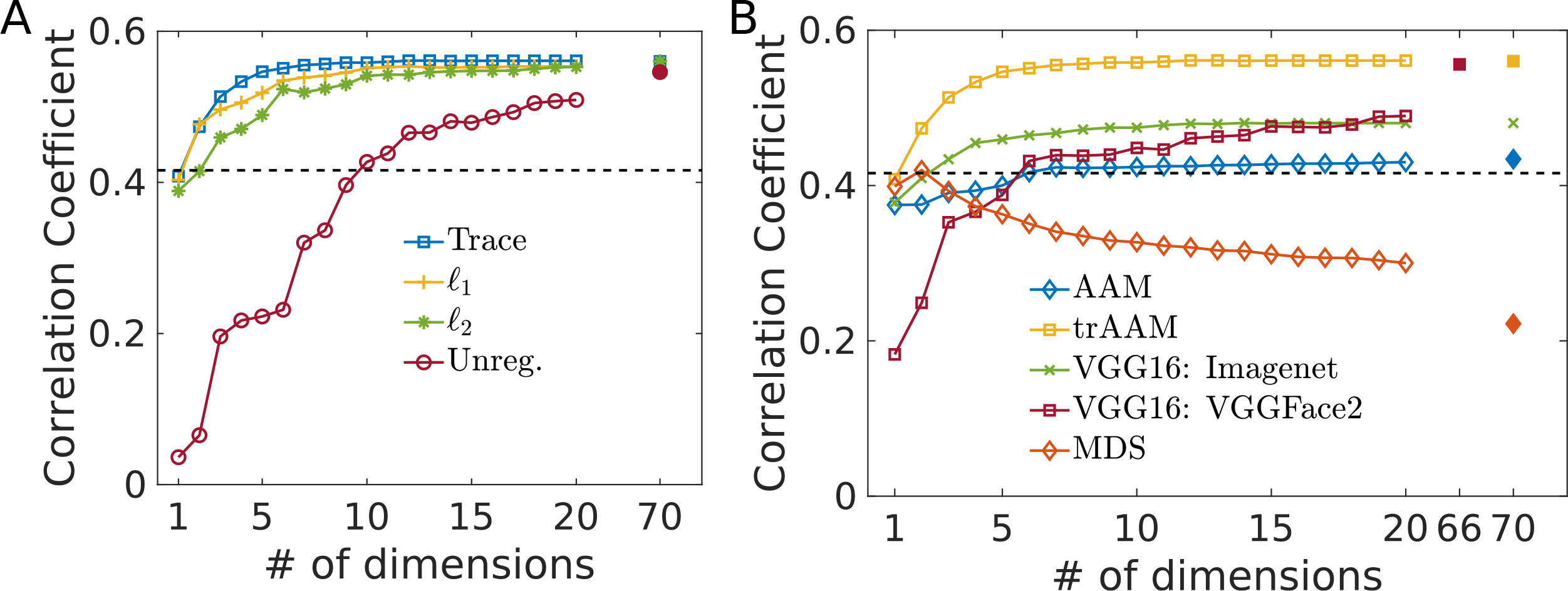
A. Effect of regularization on AAM representation. B. Evaluation of various representations; here VGG16 representations correspond to their trace regularized transformed representations. A, B evaluated on validation data (train:validation:test=8:1:1).

In addition to AAM, we also use deep neural networks to initialize the face space (see Methods). We use VGG16 (Simonyan & Zisserman, 2014) trained on ImageNet (general object categorization), the best known deep neural network representation for supporting a linear model of human social trait judgement of faces (Song et al., 2017); we also include VGG16 trained on VGGFace2 (face recognition) (Cao, Shen, Xie, Parkhi, & Zisserman, 2018). Both of these neural networks achieve much worse performance (untransformed: $r_{test}^{\text{VGG16:}\;\text{Imagenet}}=0.1$, $r_{test}^{\text{VGG16:}\;\text{VGGFace}2}=0.31$; transformed: $r_{test}^{\text{VGG16:}\;\text{Imagenet}}=0.46$, $r_{test}^{\text{VGG16:}\;\text{VGGFace}2}=0.53$) than transformed AAM, when only a dozen or so features are included, though they are substantially improved from their untransformed representations; asymptotically, VGG16 trained on VGGFace2 does a comparable job to transformed AAM (Figure 2B) – it is interesting to note this model cannot efficiently capture similarity judgments even under trace regularization. We also include a version of MDS (see Methods) for comparison. MDS is comparable to human c.c. with two features, though much worse than computer vision-based algorithms, but its performance steadily deteriorates with more features, reflecting data insufficiency in the absence of an image model.

It is notable that the regularized methods do much better than the c.c. between human ratings on the same image. Human c.c. might have been expected to be a cap on performance, but because human ratings both suffer from within-subject noise, and inter-subject inconsistency, as well as other possible violations of a metric space (e.g. violation of the triangle inequality), one person’s rating can be a rather poor predictor of how others will rate the similarity of a face pair; our algorithm can outperform this measure on a novel face because it knows where each face “lives” in the face space relative to other faces, and thus extrapolate from neighboring faces’ data to estimate the distance between two new data points.

### Dimensionality of Human Similarity Judgment Space.

Among the three types of regularization, we anticipate that trace regularization should be particularly effective in finding a small set of features. Figure 2A shows that this is indeed the case. Trace-regularized AAM achieves near-asymptotic performance with many fewer features (most important features first, as indexed by the scaling factor in the transformed space) than *ℓ*_1_- and *ℓ*_2_-regularized AAM. Using only the first 8 features achieves nearly as good of dissimilarity prediction performance (*r* = 0.557) as using all features (*r* = 0.561), while using the first 12 features (*r* = 0.561) is indistinguishable from using all features. Due to the overall superiority of the trace-regularized AAM method in capturing human similarity judgments, we primarily focus on this model in the remainder of the paper (we also sometimes refer to it simply as transformed AAM).

### Race- and Gender-Related Features in Human Similarity Judgment.

**Figure 3:**
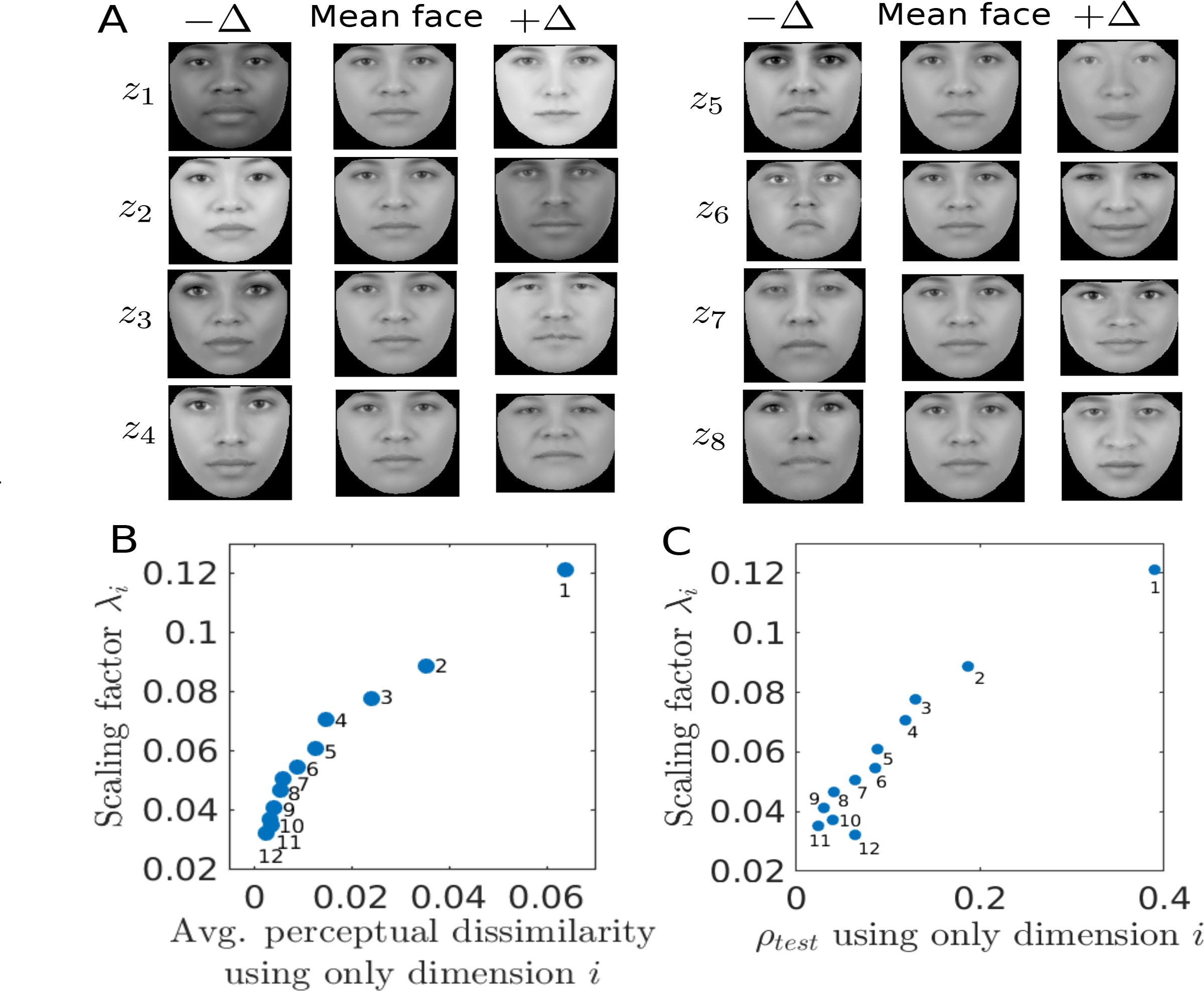
Transformed AAM features. A. Synthetic faces along each of the first 8 features (largest eigenvalues of **W**). The stepsize in each direction, **Δ**, is constant, so that every left/right face compared to the middle face evokes the same amount of perceptual dissimilarity as predicted by the model. B. Scaling factors vs average model predicted perceptual dissimilarities in trAAM along each dimension. C. Scaling factors vs c.c between model predicted and actual dissimilarity scores on test data.

Figure 3A shows synthetic faces generated along each of the first 8 features of the transformed AAM space (ordered by descending value of their scaling factors). Note that the scaling factor of a dimension is indicative of its perceptual importance – Figure 3B shows that the average perceptual dissimilarity projected along each dimension (quantifying the average importance of this dimension relative to the overall dissimilarity score) is monotonically related to the scaling factor. All the features appear to be holistic rather than parts-based, and demographic information such as race and gender is clearly present in the first few coordinates, although other more subtle, structural features are also apparent among these featural dimensions. To assess the importance of race- and gender-related features, we consider the average perceptual dissimilarity between subgroups. We note the average model-predicted dissimilarity score between the average male and female faces (0.50), between black and white faces (0.63), between Asian and black faces (0.57), between Asian and Hispanic faces (0.50), and between Asian and white faces (0.57) are all quite substantial, given that the empirical dissimilarity scores are normalized to have a maximal value of 1 and a minimal value of 0 (see Figure 1F for histogram). To quantify this more precisely, we consider the 4D subspace of X spanned by the axis that differentiates male and female faces (using linear-discriminant analysis, or LDA), and the 3D LDA subspace that best linearly discriminates among the four racial groups. We fit a linear transformation **W** within only this subspace – we find that the c.c. between this model-predicted dissimilarity and human-reported dissimilarity on held-out face pairs is *r* = 0.44, or 81% of the performance of using the full model. This indicates race- and gender-informative features figure prominently but not exclusively in human dissimilarity judgments. However, we note that this measure may be somewhat inflated, as the trace regularization suppresses the importance of other features that might also be good at differentiating individual faces but do not add much extra value – in the absence of these race- and gender-informative features, those other features may be able to at least partly make up for the lost capacity and thus achieve c.c. much higher than 19% of the full model.

### Face Space: Beyond Similarity Judgments.

**Figure 4:**
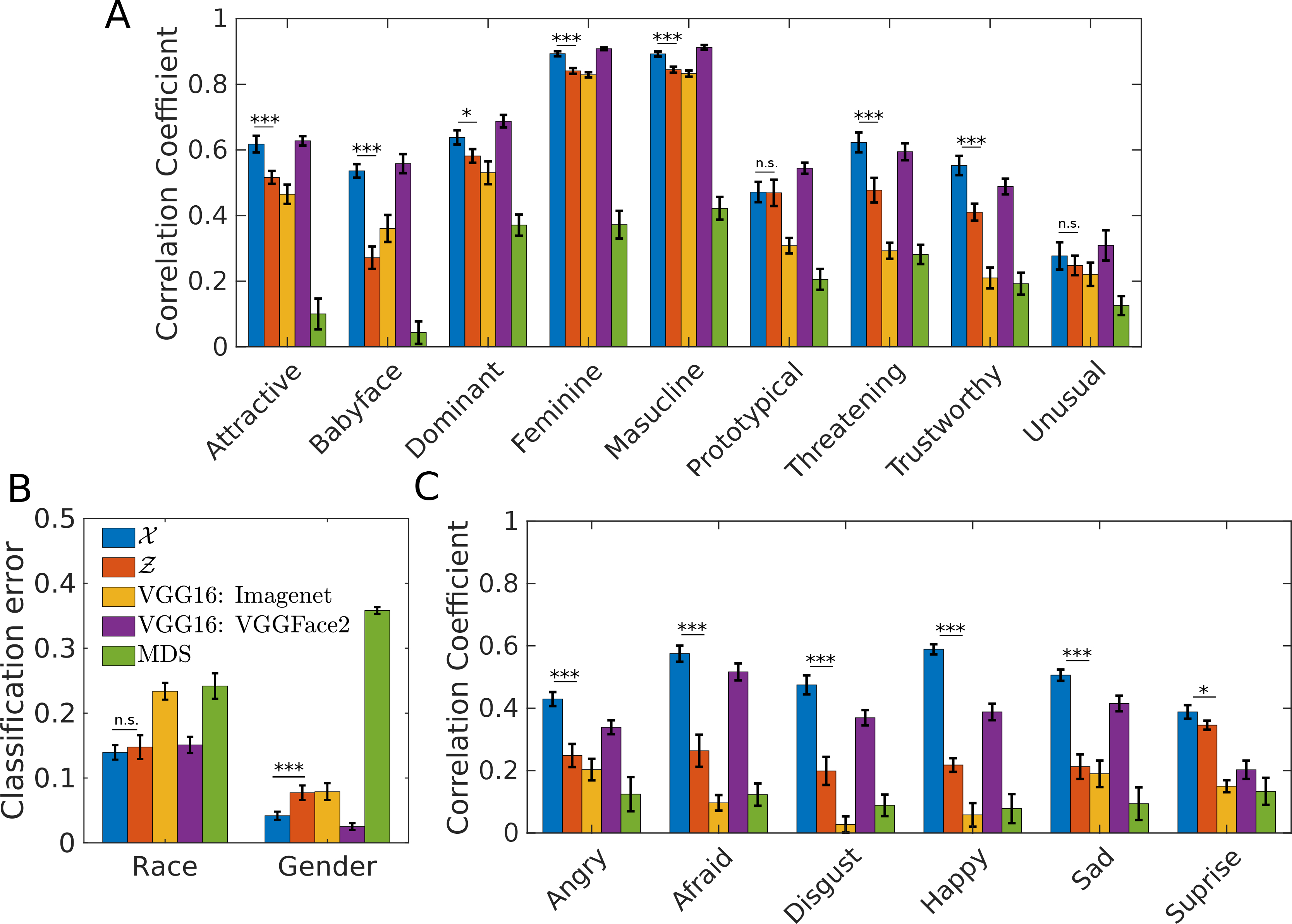
A. Social trait prediction. B. Race and gender classification error. C. Emotion ratings prediction. All error bars are SEM over 10-fold CV. Race and gender labels, human ratings of social and emotion traits are from CFD (Ma et al., 2015). n.s.: not significant at α= 0.05,∗ : *p* < 0.05,∗∗ : *p* < 0.01,∗∗∗ : *p* < 0.001; one-sided, paired two-sample *t*-test.

Implicit in the concept of a similarity-based “face space” is that features important for similarity judgments also support all other kinds of face-related processing (Valentine, 1991; Valentine, Lewis, & Hills, 2016), such as race and gender categorization, social trait perception, and affective judgments (Guan et al., 2018). Using linear modeling (LDA on categorical discrimination and linear regression on continuous predictions), we can compare how well using only the similarity-relevant features (first 8 dimensions of the transformed AAM, denoted as Z) compares to the original AAM space X, in performing other kinds of tasks. For comparison, we also include VGG16 (trained on either ImageNet or VGGFace2), and MDS. We find that X is better or comparable to both deep neural nets and MDS on all tasks (Figure 4A: social trait perception, Figure 4B: race and gender classification, Figure 4C: affect judgements). Compared to X, Z does slightly worse on social trait perception, similarly on race and slightly worse on gender, and considerably worse on all affective judgments except for “surprise.” The general tendency of X doing slightly better than Z indicates that certain features unimportant for similarity judgment play a significant role in supporting the other face-based tasks, in particular affective judgments. These results suggest that, in general, it is inadequate to use only similarity judgments to reconstruct the psychological face space, if the goal is to study also other aspects of human face processing.

## Methods

### Data Collection.

We collected human similarity judgments on pairs of face images through Amazon Mechanical Turk. The stimuli were 595 neutral-expression face images from the Chicago Face Database (CFD) (Ma et al., 2015), comprising 109 (East) Asian (57 female), 197 black (104 female), 108 Hispanic (56 female), and 181 white (90 female) faces. We randomly sampled pairs of images to produce 23,400 unique pairs, which were rated by 682 raters to produce 138,533 ratings in total. Participants rated the similarity of a pair of face images on a Likert scale from 1 (maximally dissimilar) to 9 (maximally similar); image presentation order was randomized, and subjects rated each image pair twice to counter within-subject variability (Vul & Pashler, 2008; Steegen, Dewitte, Tuerlinckx, & Vanpaemel, 2014). To identify non-attentive participants, we included a catch question, where subjects had to indicate if two identical images were the same or not.

### Participant Inclusion/Exclusion Criteria.

86 raters who failed the catch question were excluded. 4 participants who rated far fewer pairs (< 30) than the other participants (> 200 pairs) were excluded. We also excluded (15) participants whose c.c. of ratings versus other raters on the same images were at least two standard deviations below population mean. We also excluded (32) participants whose response entropy was at least two standard deviations below population mean. Included in the analysis are 111,893 ratings from 551 participants on 22,500 unique pairs of images (comprising 12.73% of the total possible pairs).

### Conversion of Similarity to Dissimilarity Measures.

To relate similarity ratings to distances in the face space, we first convert similarity into dissimilarity scores. Let $s_{\left(i,j\right)}^{r}$ denote the similarity rating for images *i* and *j* from participant *r*; we convert it to dissimilarity as $d_{\left(i,j\right)}^{r}=10-s_{\left(i,j\right)}^{r}$. We then normalize it for each participant *r*, ${\tilde{d}}_{\left(i,j\right)}^{r}=\frac{d_{\left(i,j\right)}^{r}-\min_{i,j}d_{\left(i,j\right)}^{r}}{\max_{i,j}d_{\left(i,j\right)}^{r}-\min_{i,j}d_{\left(i,j\right)}^{r}}$. For each image pair (*i, j*), we average the normalized dissimilarity ratings to produce an average score ${\bar{d}}_{\left(i,j\right)}=\sum_{r}{\tilde{d}}_{\left(i,j\right)}^{r}$. In the main text, we simply refer to the average dissimilarity score as *the dissimilarity score*.

### Computer Vision Representation: AAM.

AAM is a well-established machine vision technique that reconstructs images well, generates realistic synthetic faces (Edwards, Cootes, & Taylor, 1998), and appears to have neural relevance (Chang & Tsao, 2017). AAM consists of *shape features*, or the (x,y) coordinates of a set of consistently defined landmarks (e.g. contours of eyes, noise, lips), and *texture features*, or the grayscale pixel values of a warped version of the image after aligning the landmarks to the average landmark locations across the data set. We train AAM using faces from both CFD and 2222 US adult face images from Google Images (Bainbridge, Isola, & Oliva, 2013). We use the free software Face++ ^1^ to labels 83 landmarks on each face. We apply combined PCA to all the shape and texture features, yielding a 70-dimensional representation that captures 98% of the variance.

### Computer Vision Representation: VGG16.

VGG16 is a deep Convolution Neural Network (CNN) used for general object recognition (Simonyan & Zisserman, 2014). It has been trained using the Imagenet dataset containing 1000 categories of objects, totalling 1.3 million images (Russakovsky et al., 2015; Deng et al., 2009). Once a face image used in our similarity judgment task is fed into this network, we use the response in the penultimate layer as the image’s initial representation. We also use the same architecture trained on VGGFace2 (Cao et al., 2018) (face recognition). We then perform PCA on extracted features to reduce dimensionality: we retain features capturing 98% of the variance in the CFD dataset (Imagnet-100 PC’s, VGGFace2–66 PC’s).

### Metric Learning.

We assume human dissimilarity scores are noisy versions of *f*(**x**_*i*_,**x**_*j*_), where *f*(**x**_*i*_,x_*j*_) = (**x**_*i*_ − **x**_*j*_)^**⊤**^**W**(**x**_*i*_ −**x**_*j*_)+*b*, where **W** is constrained to be positive semidefinite (PSD; i.e. non-negative eigenvalues) and *b* ≥ 0 is a constant offset (*b* has a fitted value of 0.47 in our main model, trace-regularized AAM). Since **W** is PSD, it can be diagonalized as **W** = **U**^**⊤**^**ΛU**, where **U** is an orthogonal transformation and **Λ**= diag(λ_1_, …, λ_*n*_), where λ_*i*_ ≥ 0 are the eigenvalues of **W** ^2^. Constraining **W** to be a diagonal matrix means that the new coordinate system consists of rescaling the original axes, but no rotations are allowed. Allowing **W** to be any PSD matrix means the original basis vectors can be rotated and reflected (**U** consists of the eigenvectors of **W** and specifies the directions of the new basis vectors), and then multiplicatively scaled by the square root of the entries of **Λ** (the eigenvalues of **W**) to arrive at the new basis vectors. Allowing **W** to have 0 as an eigenvalue means that some featural dimensions in the transformed space are allowed to shrink to nothing and thus play no role in perceived dissimilarities.

We then aim to minimize prediction error while regularizing the *ℓ*_1_ or *ℓ*_2_ norm of **W**. To implement *ℓ*_1_ and *ℓ*_2_ regularization, we minimize the following objective function, denoting **x**_(*i,j*)_ =(**x**_*i*_ −**x**_*j*_) and subject to **W** ⪰ 0, *b* ≥ 0,

$$\underset{W,b}{\min}\sum_{i,j}{\left({\bar{d}}_{\left(i,j\right)}-x_{\left(i,j\right)}^{⊤}Wx_{\left(i,j\right)}-b\right)}^{2}+\alpha \|W{\|}_{p}$$

where *p* = 1 corresponds to *ℓ*_1_ norm, and *p* = 2 corresponds to *ℓ*_2_ norm. No regularization can be considered a special case (α= 0). This is a convex optimization problem, and can be solved via semi-definite programming (we use CVX (Grant & Boyd, 2014, 2008)). We set the value of the regularization coefficient α using line search and evaluation on held-out validation data (choose α that gives the best dissimilarity prediction on the validation set).

To find a small set of interpretable features, we need to suppress the dimensionality of **W** (non-zero eigenvalues). *ℓ*_1_ and *ℓ*_2_ regularization are inappropriate because in the former case, both the rotation (**U**) and the scaling (**Λ**) components are restricted, while in the latter, the regularization term is not effective at encouraging the eigenvalues to go to zero, $\|W{\|}_{2}=\sqrt{\mathrm{tr}\left(W^{⊤}W\right)}=\sqrt{\mathrm{tr}\left(\left(U^{⊤}\Lambda U\right)\left(U^{⊤}\Lambda U\right)\right)}=\sqrt{\sum_{i}\lambda_{i}^{2}}$. To reduce the number of basis vectors (non-zero eigenvalues), we penalize the sum of the eigenvalues, or trace(**Λ**) = trace(**W**), resulting in another convex optimization problem (subject to **W** ⪰ 0, *b* ≥ 0):

$$\underset{W,b}{\min}\sum_{i,j}{\left({\bar{d}}_{\left(i,j\right)}-x_{\left(i,j\right)}^{⊤}Wx_{\left(i,j\right)}-b\right)}^{2}+\lambda \mathrm{trace}(W).$$

### Multidimensional Scaling.

We utilize a version of MDS known as classical MDS (Torgerson, 1965), which attempts to find coordinates of points in an abstract multidimensional space, such that the inter-point dissimilarities are well-preserved when modeled as Euclidean distances in this space. Consider a graph G with faces images as nodes, and an edge exists between nodes *i* and *j* with length ${\bar{d}}_{\left(i,j\right)}$, if the training dataset contains the dissimilarity score for this pair. Since MDS requires dissimilarities between every pair of images to learn a representation, we estimate the missing distances (edges) as the *shortest* path (sum of edge lengths) between two nodes in G (Shang, Ruml, Zhang, & Fromherz, 2003). Once all pairwise distances have been specified (or estimated), we then run classical MDS to obtain coordinates for all the data points. We also implemented alternative ways to estimate the missing pairwise distances, as well as variants of MDS, but as they achieved poorer similarity prediction performance on held-out data, we will not discuss them further.

## Discussion

In this paper, we presented a novel way of modeling the psychological face space, by first initializing it with a computer vision representation, then linearly transforming it to reproduce human dissimilarity ratings of faces as well as possible. Methodologically, while our broad approach is related to transfer learning (Razavian, Azizpour, Sullivan, & Carlsson, 2014; Peterson, Abbott, & Griffiths, 2016), we also presented a novel regularization method, that allowed us to make a rather surprising scientific finding: only the 8–12 most important facial features of our model are sufficient to achieve nearly the capacity of the full model to model human face processing, suggesting that the psychological face space may be rather low-dimensional.

By construction, our approach overcomes many of the critical limitations of a common approach in this field (Dailey et al., 1999; Nestor et al., 2016; Nishimura et al., 2009; Shepard, 1962; Torgerson, 1965), namely MDS, by being more interpretable, invertible, generalizable, and data efficient. In addition, we showed that while this method is far better at modeling both dissimilarity judgments and human performance on other face-based tasks (categorizing gender and race, assessing social traits, rating emotional expressions), compared to MDS. However, using only the similarity-relevant features does not work as well as also including the orthogonal features, especially for affective judgments. This scientific finding is at odds with an implicit assumption about human face representation in the psychology literature (Valentine, 1991), which, by attempting to reconstructing the full psychological face space using only pairwise similarity judgments, assumes that features important for these judgments are also sufficient for all other face-based tasks.

Another interesting finding is that AAM provides a better initial representation than convolutional deep neural networks trained on both object recognition and face recognition, both for similarity judgments and for other human face-based tasks. We find that VGG16 trained on face recognition (VGGFace2) comes the closest, but is highly inefficient in terms of the number of features it needs to capture similarity judgments (despite having the same trace regularization applied to both). An interesting line of future research would be to consider various unsupervised learning variants of deep neural nets, which may not only learn psychologically relevant features, but also incorporate a decoder model that can generate synthetic images to help visualize/interpret the latent feature space. In particular, adopting techniques that explicitly incorporate inductive biases about shape and texture into the architecture seem promising (Shu et al., 2018; Nguyen-Phuoc, Li, Theis, Richardt, & Yang, 2019).

## Footnotes

1
https://www.faceplusplus.com

2 Note that we’re actually modeling the Euclidean distance squared as the the dissimilarity score, as we found this to be empirically better. We may interpret this as modeling a fixed transformation or “link” function of the dissimilarity score (specifically, ${\left({\bar{d}}_{\left(i,j\right)}-b\right)}^{0.5}$) as the Euclidean distance. We experimented with many other monotonic link functions but did not obtain better results, and will not discuss them here.
